# Aligning opportunity cost and net benefit criteria: the health shadow price

**DOI:** 10.3389/fpubh.2024.1212439

**Published:** 2024-03-06

**Authors:** Simon Eckermann

**Affiliations:** School of Health and Society, University of Wollongong, Wollongong, NSW, Australia

**Keywords:** opportunity cost, net benefit, threshold value, health shadow price, budget constrained optimization, allocative efficiency, displacement efficiency

## Abstract

Given constrained healthcare budgets and many competing demands, public health decision-making requires comparing the expected cost and health outcomes of alternative strategies and associated adoption and financing actions. Opportunity cost (comparing outcomes from the best alternative use of budgets or actions in decision making) and more recently net benefit criteria (relative valuing of effects at a threshold value less costs) have been key concepts and metrics applied toward making such decisions. In an ideal world, opportunity cost and net benefit criteria should be mutually supportive and consistent. However, that requires a threshold value to align net benefit with opportunity cost assessment. This perspective piece shows that using the health shadow price as the ICER threshold aligns net benefit and opportunity cost criteria for joint adoption and financing actions that arise when reimbursing any new strategy or technology under a constrained budget. For an investment strategy with ICER at the health shadow price *Bc* = 1/(1/*n* + 1/*d*-1/*m*), net benefit of reimbursing (adopting and financing) that strategy given an incremental cost-effectiveness ration (ICER) of actual displacement, *d*, in financing, is shown to be equivalent to that of the best alternative actions, the most cost-effective expansion of existing programs (ICER = *n*) funded by the contraction of the least cost-effective programs (ICER = *m*). Net benefit is correspondingly positive or negative if it is below or above this threshold. Implications are discussed for creating pathways to optimal public health decision-making with appropriate incentives for efficient displacement as well as for adoption actions and related research.

## 1 Introduction

Optimizing health outcomes from a constrained public health system budget requires decisions about which programs and strategies to support through reimbursement when investing in or allocating budgets and resources. Opportunity cost and net benefit criteria have been key concepts and metrics applied to inform budget-constrained optimal health system decision-making. This review paper introduces opportunity cost (section 2) and net benefit criteria (section 3) and shows how they are aligned using the health shadow price (section 4) as a threshold value for net benefit (section 5). This finding and its implications are discussed in section 6 in relation to creating pathways for optimal resource allocation and investment decisions with joint adoption and financing actions, but also to incentivize research in identifying the best expansion and contraction of programs with constrained budgets.

## 2 Opportunity cost

Health systems with constrained budgets or resources and unconstrained population health need to make allocative decisions across alternative strategies. Given constrained resources and funding, the opportunity cost of any resource use or reimbursement/investment decision and associated action/s (adoption and financing with reimbursement), is the forgone value of outcomes from the best alternative use or choice and associated set of actions (1–5).

As a result, all constrained healthcare system funding or resource allocation processes face opportunity costs with each reimbursement or investment decision. That is, the value of the best alternative investment or reimbursement choice with adoption and necessary associated financing actions under a constrained budget.

## 3 Net benefit criteria

The net benefit is a metric that combines the joint consideration of effects and costs (resource use and their prices) to compare the relative value less cost of strategies, decisions, or action/s (6–9). For the simplest case of two strategy comparisons, the incremental net benefit (INB) of a strategy relative to a comparator is:


(1) INB=λΔE−ΔC

Where λ is the threshold value for effects and ΔE and ΔC are incremental effects and costs. Positive INB implies invest, while 0 or negative INB implies don’t invest. Net benefit metrics have a series of advantages over incremental cost-effectiveness ratios (ICERs) (9–17). These include being well ordered when the effect is 0 or changes sign around 0, having an opposite sign consistent with the implications when a strategy is dominated or dominates, and reflecting the degree of any such dominance (9). In addition, additive separability of net benefit (although not ICERs) (10) implies that multiple strategies or multiple domains can be consistently compared relative to any comparator based on maximizing net benefit (NB = λE−C) or equivalently minimizing net loss (NL = λDU + C) under the net benefit correspondence theorem (11–17). DU in the net loss metric simply represents any effect/s (E) in net benefit framed from a disutility perspective (e.g., mortality vs. survival, morbidity vs. no morbidity, QALYs lost vs. QALYs gained, etc.).

## 4 The health shadow price

Given a fixed or constrained budget, the health shadow price represents the critical or threshold value at which the health outcome from any reimbursement decision (adoption and financing action) equates with the best alternative adoption and financing actions (4, 5). The best alternative adoption and financing actions are to implement the most cost-effective expansion of existing programs (ICER = *n*, e.g., $5,000/QALY) funded by reducing the least cost-effective programs (ICER = *m*, e.g., $ 1 million/QALY).

Thus, with a constrained budget, for any given investment I with actual displacement (ICER = *d*, e.g., $20,000/QALY) the health shadow price ( $B_{C}$ ) for any given strategy is found by setting the net health return from reimbursing (adopting and financing) that strategy for any given investment amount I , $I/B_{C}-I/d$ , as equal to that from the best alternative joint adoption and financing actions I/n−I/m.

Consequently, solving


$$\frac{I}{B_{c}}-\frac{I}{d}=\frac{I}{n}-\frac{I}{m}$$

results in:


$$\frac{1}{B_{c}}=\frac{1}{n}+\frac{1}{d}-\frac{1}{m}$$

or


(2) $$B_{c}={\left(\frac{1}{n}+\frac{1}{d}-\frac{1}{m}\;\right)}^{-1}$$

The subscript c denotes that the health shadow price (Equation 2), like opportunity cost, corresponds to the economic context of the best alternative actions within a constrained budget (4, 5, 18, 19). Explicitly the context for making budget constrained reimbursement (joint adoption and financing) decisions. That is the context of actual displacement (ICER= *d*), and best alternative expansion (ICER= *n*) and contraction (ICER= *m*) actions.

For example, if *n* = $5,000/QALY, *d* = $20,000/QALY, and *m* = $1,000,000/QALY, then


$$\begin{array}{l} B_{c}=\frac{1}{\frac{1}{5000}+\frac{1}{20000}-\frac{1}{1000000}}=\\ \frac{1}{0.0002+0.00005-0.0000001}=\\ \frac{1}{0.0002499}=\$4001.60/QALY \end{array}$$

## 5 Incremental net benefit consistent with opportunity cost

If we conduct an incremental net benefit assessment of an investment or reimbursement amount I relative to the best alternative investment or reimbursement of I (adoption and financing actions) using the health shadow price as a threshold value then from Equation 1 and Equation 2 we have:


$$INB=B_{C}\Delta E-\Delta C$$

Now for cases of interest where ΔE and ΔC are both positive, i.e., in the NE quadrant, then INB using the health shadow price as a threshold value is only positive where ${Β}_{c}$ ΔE−ΔC > 0 and hence ΔC/ΔE< ${Β}_{c}\;$ or negative where ${Β}_{c}$ ΔE−ΔC < 0 and hence ΔC/ΔE> ${Β}_{c}.$ QED.

Hence, using the health shadow price, ${Β}_{c}\;$ as the threshold value for effects aligns INB with the same decisions as comparing the investment ICER with the health shadow price—which is derived from and represents the opportunity cost of budget-constrained investments.

## 6 Discussion: implications for optimal research, resource allocation, and investment

In sections 2–5 we have shown that using the health shadow price as the critical threshold value for incremental net benefit appropriately results in equivalent net benefit between:

- Any new budget constrained investment (I) in a strategy with ICER equal to the health shadow price funded by displacement (ICER = *d*) and;
- The best alternative health system adoption and financing actions for the same budget constrained investment I.

Furthermore, if the strategy has a lower or higher ICER than this, then its INB is appropriately correspondingly positive or negative.

Importantly this means that for any investment with a constrained budget using the health shadow price as the net benefit threshold value enables net benefit to represent the same rule as opportunity cost.

If the budget was not constrained then investment would only have an adoption action, for which the best alternative action is the most cost-effective expansion of existing programs (ICER = *n*), which would be the appropriate threshold value. Indeed, this is also the appropriate threshold value for the health shadow price if displacement is efficient (*d* = *m*), which is then given by:


$$B_{c}={\left(\frac{1}{n}+\frac{1}{d}-\frac{1}{m}\;\right)}^{-1}=\frac{1}{\frac{1}{n}}=n$$

In response to experiences in the United Kingdom during the 1990 and 2000s where many services were displaced by the mandated use of medications such as Herceptin (20) different ICER thresholds for displaced services were proposed as a threshold value for INB assessment (21–27). Four different displaced service thresholds were proposed:

- The least cost-effective current program, assuming that this is the program that is actually displaced to finance the additional costs of the new technology (22).
- The least cost-effective program, regardless of whether or not it is displaced (23).
- The ICER of the services actually displaced to finance that technology regardless of the ICER of that displaced service relative to other services (24–26).
- The average ICER of historically displaced NHS services (21, 27).

Strictly speaking, these four definitions would only coincide if displacement had been and remained currently efficient (*d* = *m*). Nevertheless, the health shadow price makes clear the proposed use of *d* or any displaced service as a threshold in all its guises (18, 19):

- Conflates adoption and financing actions—equates the threshold value in expansion with that in contraction (25).
- Arises only at a singular point where there is perfect allocative efficiency, but does not provide a pathway to get there.
- Most importantly, it denies the true opportunity cost of reimbursing (adopting and funding) new technologies—the most cost-effective expansion of existing health system interventions funded by displacement of the least cost-effective interventions.

Sendi et al. (21) advocated the average ICER of displaced services as a second-best alternative threshold value to the shadow price of the budget constraint reflecting the shadow price per unit of effectiveness in the absence of a market. They emphasized that this, like the shadow price, is a function of program size (5, p.82). Later, theoretical arguments or assertions were made for displaced service-related ICERs representing the shadow price of the budget constraint (22–27), explicitly stated in Griffin et al. (25, p.24) as the ICER of actual displaced services, *d*, with a two-part argument:

- “Identifying marginal programs that would be displaced and quantifying their cost and health outcomes determines the shadow price of the budget constraint” [SIC].
- “The incremental cost per QALY gained of new treatments are commonly compared to some stated threshold λ, which should in principle represent the inverse of the shadow price of the budget constraint” [SIC].

These two parts were combined to argue that any new treatment should be reimbursed if the incremental health offered by the new treatment option exceeds the health foregone with the displacement of marginal programs (25, p.24).

However, in the context of market failure and allocative inefficiency characteristic of the health care system, displaced service definitions in general, and this two-part argument in particular, conflate shadow price in expansion (maximum unit of effect gained as a result of relaxing a constraint by one unit at the margin) (28), with notions of shadow price in contraction (minimum loss when one unit of a continuous resource is withdrawn) (29). Combined, the two parts misrepresent opportunity cost as the actual loss of displacement when the budget is reduced, rather than the highest value alternative (18). Displaced services do not represent opportunity cost, the highest value alternative. If Griffin et al. (25) had appropriately added “in expansion” in part (ii) to represent opportunity cost, it would be clear that this is different from any notion of the shadow price of the budget constraint “in contraction” in part (ii). The only situation in which shadow prices in expansion and contraction can coincide is at the single point of complete allocative efficiency (*n* = *m*) and indeed of displacement efficiency (*d* = *m*) and hence *n* = *m* = *d=* $B_{c}$ .

Empirically, no health system internationally can claim to beat the point of allocative efficiency. Consider the case of the United Kingdom which was ranked in 2014 by the Commonwealth Fund (30) as having the most efficient health system of high income OECD countries. Despite this international standing, the 2013 evidence from Claxton et al. (27) of the ICER for best expansion (*n* = 2,000 pounds per QALY), contraction (*m* = 2.73 million pounds per QALY) and displacement (*d* = 12,713 pounds per QALY) indicated substantial allocative and displacement inefficiency in practice with *n* < *d* < <*m* (19, 31). This resulted in a health shadow price [following Equation (2)] with 2013 United Kingdom evidence (27) of:


$$\begin{array}{l} B_{c}=\frac{1}{\frac{1}{2000}+\frac{1}{12713}-\frac{1}{2730000}}=\\ \frac{1}{0.0005+0.0000787-0.000000366}=\\ \frac{1}{0.0005783}=1,729\;\mathrm{pounds}/\mathrm{QALY} \end{array}$$

Pekarsky (5, p.69-83) shows that in the context of such economic inefficiency and market failure for an input, deriving the health shadow price from existing information about the economic context than from cost benefit analysis following McKean (32) and Mishah and Quah (3). That is, the health shadow price (4, 5) as derived in section 4 above, takes into account observed allocative (*n < m*) and displacement (*d < m*) inefficiencies, rather than any notion of the shadow price of the budget constraint that assumes perfect economic efficiency.

An underlying inappropriate assumption for health care of complete allocative efficiency in the context of market failure can be traced back to the suggested use of shadow price of the budget constraint as a critical ratio for the threshold value by Weinstein and Zeckerhaus (33). They implicitly assumed complete allocative efficiency (and discrete programs) by suggesting a critical ratio of the cost per effect of the last service financed if all services are ranked and allocated up to the budget—the average shadow price.

As Pekarsky (5, p. 83) surmises: “…health economic focus on the shadow price of the budget constraint…can lead to the following catch 22: we cannot find this shadow price until economic efficiency is achieved, and we cannot achieve economic efficiency until this price is found” [*SIC*]. Importantly, the health shadow price (4, 5) creates the appropriate incentives for evidence on the best expansion and contraction of current programs to enable it (18, 19). This meets the imperative to “find a shadow price for health effects that will improve economic efficiency rather than being conditional on economic efficiency” [*SIC*] (5, p. 83). The only point where the health shadow price (4, 5) coincides with the ICER for actual displaced services, *d*, is at the point of complete allocative (and displacement) efficiency; and because *n*=*m*=*d*=B_*c*_ only at that singular point. However, using *d* as a threshold value does not provide a pathway to get there, whereas the health shadow price does (18, 19). That is, the coincidence of the shadow price in expansion and contraction at the single point of allocative efficiency does not provide a pathway to get there from any point of allocative inefficiency. Certainly not when using a displaced service ICER threshold such as d, which is only equal to the health shadow price at the single point of complete allocative efficiency. Moreover, considered dynamically over time with allocative inefficiency (*n* < *m*) if displacement were efficient (*d = m*) then new technologies or strategies priced up to a threshold value of *d* would be next in line to be displaced and would face additional reversal costs not accounted for in ICER calculations as the new technology cycles through. Hence, as highlighted in Eckermann and Pekarsky (18) use of *d* as a threshold with appropriate consideration of reversal costs can easily lead to health outcomes from constrained budgets declining over time, particularly if displacement is efficient.

Now, let us consider if comparison of the reimbursement of an investment were not with the best alternative action/s to reflect opportunity costs, but rather with the second best objective of improving the (short term) net benefit from combined adoption and displacement decisions. Then, with a fixed budget the ICER from adopting new programs could be considered relative to that of actual displaced services (21). However, to the extent that displacement is efficient (*d* approaches m rather than *n*) this creates a straw man for comparing adoption (even without a budget constraint), let alone if joint adoption and financing actions are appropriately considered together with the health shadow price (18).

The alignment of net benefit with opportunity cost for budget-constrained investment or reimbursement (adoption and financing actions) that arises with the use of the health shadow price ( $B_{C}$ ) as the ICER threshold value does not occur with *d* (the ICER of displaced services). That is clear noting that generally *B*_*c*_ ≤ *n* ≤ *d* ≤ *m*. They only coincide at the single point of complete allocative and displacement efficiency, which no health system internationally satisfies, with *n* = *m* = *d=* $B_{c}$ at this point alone.

This implies that using a threshold value of *d* for net benefit assessment biases against better use of existing programs or technologies and in favor of pricing new technologies above opportunity cost, given the best alternative reimbursement actions (adoption and financing) that the health shadow price represents.

More generally, in the absence of appropriate incentives created by the health shadow price research decisions are also biased toward evidence for new technologies. On the other hand, there are disincentives for evidence or indeed lack of evidence for unpatented or unpatentable strategies (4, 5, 18, 19). In particular, this would bias against public health strategies such as those for health promotion across the life course (34). Those strategies include community programs that support integrated movement from early childhood to youth, adulthood and older adulthood (35–39); successful ageing (40–44) or rehabilitation and palliative care services (16, 19, 45). The same types of services that were displaced to accommodate mandated Herceptin use in the United Kingdom highlighted in the 2000s (20).

It could be argued that pharmaceutical and device manufacturing companies are only responsible for adoption and should not be penalized for inefficient displacement (*d < m*). However, the reality of a constrained budget is that adoption and financing actions (and associated research) for any reimbursement (investment) decision naturally need to occur together. More generally optimal cycles of joint research, reimbursement and regulatory decision making are required to optimize budget-constrained health system outcomes in any jurisdiction and globally (16, 46). Where research funding is biased in favor of new technologies and against unpatentable technologies or programs (e.g., community programs without vested interests), a pathway to optimization requires public policy incentives for research evidence and better use of existing programs and technologies in adoption and displacement (4, 5, 18, 19). Ideally, processes supporting optimal cycles of research, reimbursement and regulatory decision making (16) that systematically reflect and create an imperative for that pathway.

Consequently use the health shadow price as a threshold value for net benefit assessment is key to creating appropriate incentives for research evidence to support displacement or contraction and for appropriate adoption or expansion actions (4, 5, 18, 19). The use of the health shadow price makes it clear that there are joint adoption and financing actions and associated research requirements for any reimbursement/investment decision with constrained budgets.

## 7 Conclusion

This paper has shown that incremental net benefit aligns with opportunity cost when the health shadow price (section 4) is used as the threshold value. At this threshold value, section 5 showed that the incremental net benefit criteria (section 3) of an investment are positive (negative) only if the health outcomes from that investment are greater (less) than the best alternative adoption and financing actions (opportunity cost section 2). Hence, using the health shadow price as the threshold value for the incremental net benefit assessment in any jurisdiction makes the same reimbursement decision as opportunity cost (section 5). As the discussion in section 6 highlighted, these findings also allow for optimization in resource allocation and investment decisions and appropriate incentives for research in addition to optimal adoption and financing actions.

## Data availability statement

The original contributions presented in the study are included in the article/supplementary material, further inquiries can be directed to the corresponding author.

## Author contributions

SE conceived and designed the paper and is accountable for all aspects of the work in ensuring that questions related to the accuracy or integrity of any part of the work are appropriately investigated and resolved.
